# Association between MICA polymorphisms, s-MICA levels, and pancreatic cancer risk in a population-based case-control study

**DOI:** 10.1371/journal.pone.0217868

**Published:** 2019-06-05

**Authors:** Guillaume Onyeaghala, John Lane, Nathan Pankratz, Heather H. Nelson, Bharat Thyagarajan, Bruce Walcheck, Kristin E. Anderson, Anna E. Prizment

**Affiliations:** 1 Division of Epidemiology and Community Health, University of Minnesota School of Public Health, Minneapolis, MN, United States of America; 2 University of Minnesota Masonic Cancer Center, Minneapolis, MN, United States of America; 3 Department of Laboratory Medicine and Pathology, University of Minnesota, Minneapolis, MN, United States of America; 4 Department of Veterinary and Biomedical Sciences, University of Minnesota, Saint Paul, MN, United States of America; King Saud University, SAUDI ARABIA

## Abstract

**Background:**

Pancreatic tumor cells may avoid immune surveillance by releasing the transmembrane major histocompatibility complex class I chain-related A (MICA) protein in soluble form (s-MICA). We hypothesized that the presence of the A5.1 polymorphism in the MICA gene, which encodes a truncated MICA protein, is associated with higher s-MICA levels and increased pancreatic cancer risk.

**Methods:**

MICA alleles and s-MICA levels were measured in 121 pancreatic cancer cases and 419 controls. General linear regression with a log transformation assessed geometric means of s-MICA levels across MICA alleles. Unconditional logistic regression was used to calculate the odds ratio (OR) and 95% confidence intervals (CI) for pancreatic cancer associated with MICA alleles.

**Results:**

After multivariate adjustment, participants with at least one copy of the A5.1 allele versus no A5.1 allele had 1.35 (95% CI: 1.05–1.74) times greater s-MICA levels (1.65 times higher for cases and 1.28, for controls) and increased risk of pancreatic cancer (OR = 1.91, 95% CI: 1.05–3.48).

**Conclusions:**

Our study suggests higher risk of pancreatic cancer among those with the MICA A5.1 polymorphism, which may be explained by an increase in s-MICA secretion and impaired immune response.

**Impact:**

These findings provide further evidence at the genetic and molecular level of the important role of MICA in pancreatic cancer development, and may have important implications with regards to pancreatic cancer screening.

## Introduction

Pancreatic cancer is the 4th leading cause of cancer death among men and women in the U.S., with over 40,000 deaths annually[1]. Currently, there are no screening tests to detect pancreatic cancer at an early stage. [2,3] Most pancreatic cancer patients are diagnosed at an advanced stage when benefits of treatment are very limited and thus most cases have an extremely poor prognosis, with a 9% five-year survival rate[1]. Therefore, there is an urgent need for new methods for early detection and treatment of pancreatic cancer. Emerging evidence shows that the immune system plays an important role in the pathogenesis of pancreatic cancer[4,5]. Understanding specific immune system mechanisms that interact with pancreatic tumor cells could help determine high-risk groups who may benefit from screening and lead to new therapies in the future.

Immune cells such as NK cells, gamma delta (γδ) T cells, and alpha beta (αβ) CD8 + T cells can target and eliminate pancreatic tumor cells when their NKG2D (natural-killer group 2, member D) receptors bind to the major histocompatibility complex class I-related chain A (MICA) protein expressed on tumor cells[5–7]. MICA is a transmembrane protein that, in response to various cellular stresses, is expressed on the surface of epithelial cells [7–11] and, may be shed into the blood circulation in its soluble form (s-MICA)[8,12–16]. The release of MICA into circulation may lead to decreased binding affinity between NKG2D-bearing immune cells and pancreatic tumor cells, resulting in insufficient immune surveillance.

The binding affinity of MICA to NKG2D receptor on immune cells and its shedding into circulation may be modulated, in part, by polymorphisms in the MICA gene[17–19]. The MICA gene is highly polymorphic, with over 80 alleles identified to date [www.ebi.ac.uk/imgt/hla/] [7,18]. The MICA protein consists of three extracellular domains, namely α1 (encoded by exon 2), α2 (encoded by exon 3), and α3 (encoded by exon 4), a transmembrane (TM) region (encoded by exon 5) and a cytoplasmic tail (encoded by exon 6). The transmembrane domain of the MICA protein is encoded by alleles characterized by a variable number of short tandem repeat (STR) polymorphisms, consisting of 4, 5, 6, 7, 8, 9 and 10 GCT repeats, designated as A4, A5, A6, A7, A8, A9, A10 respectively[17,18]. In addition, the A5.1 allele contains an extra guanine (G) insertion after 5 GCT repeats, which causes a frameshift polymorphism leading to a premature stop codon. Compared to its non-mutated counterparts, the MICA A5.1 protein is shorter and more easily cleaved from the cell surface by the disentegrins and metalloproteases (ADAM) 10 and 17 [12,17,18].

Recent papers have demonstrated that the MICA A5.1 polymorphism modulates cancer susceptibility in several cancer types including cervical cancer[20], oral squamous cell carcinoma[21–23] and hepatocellular carcinoma[24–26]. However, no epidemiologic studies to date have evaluated the MICA A5.1 polymorphism in relation to pancreatic cancer risk. We hypothesized that the presence of the A5.1 MICA allele is associated with higher circulating s-MICA levels and increased pancreatic cancer risk. We tested this hypothesis in a population-based case-control study of pancreatic cancer in Minnesota. In addition, in an exploratory analysis, we investigated the pancreatic cancer risk associated with four other MICA-STR polymorphisms[17,18] and eight MICA single nucleotide polymorphisms (SNPs) with known associations to cancer [24,27–30].

## Materials and methods

### Study design

As described previously[31], the participants for this study were recruited between 1994 and 1998 in Minnesota. Newly diagnosed pancreatic cancer cases were recruited from all hospitals in the seven-county metropolitan area of the Twin Cities of Minnesota (i.e., Minneapolis and St. Paul) and the Mayo Clinic, where cases were restricted to those who lived in the upper Midwest[32–35]. Patients with pancreatic cancer were eligible for the study if they were 20 years of age or older, English-speaking, and gave informed consent[32–35]. Of the 460 eligible cases, 85 cases were excluded due to death before contact or the interview, 79 cases were excluded due to participant refusal, 31 cases were not invited due to physician refusal, and 7 participants could not be contacted. After those exclusions, 258 cases from the original sample participated in the study (56%).

Potential controls for the study were selected from drivers’ license lists for individuals between 20 and 64 years of age, and from US Health Care Financing Administration records for those aged 65 years and above using stratified random sampling from the seven-county metropolitan area of the Twin Cities. Controls were frequency matched to cases by age (within 5 years), sex and race. Inclusion criteria for controls were the same as those for cases, in addition to no prior diagnosis of pancreatic cancer. Of 1145 eligible controls, 676 participated in the study (59%).

Written, informed consent was obtained from all study participants prior to their interview. The protocol for this case-control study was approved by the Institutional Review Boards of the University of Minnesota and the Mayo Clinic. All study participants were interviewed in person about demographics, cigarette smoking, physical activity, dietary and alcohol intake, and medical history. Our analysis was restricted to Caucasians, who represented 96% of all study participants. Participants were asked to donate a blood sample at the time of the in-person interviews, and 30 mL of venous blood were drawn from each consenting participant. DNA was isolated by phenol-chloroform and stored at -70°C and stored until further analysis[35,36]. After excluding participants without blood samples, a total of 121 cases and 419 controls were available for the current analysis (n = 540).

### Genotyping of MICA genetic variants

For genotyping of the STR polymorphisms in the transmembrane region of the MICA gene, MICA specific PCR primers flanking exon 5 in the MICA gene were used (MICA5F, 5′CCTTTTTTTCAGGG AAA GTGC 3; MICA5R, 5′ CCTTACCATCTCCA GAAACTGC 3′)[22,37]. Samples were amplified using a multiplexed PCR approach, then indexed, pooled, and sequenced using a 2x300 bp MiSeq lane using the Illumina MiSeq Personal Sequencing platform. The resulting reads were aligned to the hg19 reference genome using the Burrows-Wheeler transform (BWA-MEM) and processed with the genome analysis toolkit (GATK) for base quality score recalibration and indel realignment[38,39]. In an exploratory analysis, eight additional MICA SNPs associated with cancer in previous studies were genotyped using the GATK HaplotypeCaller.

MICA STR genotypes were assigned by counting the number of sequence reads from known alleles (A4, A5, A6, A7, A9, A10, and A5.1) seen in sequence reads overlapping the STR region. Samples where a single corresponding allele was detected were called homozygous and samples where two corresponding alleles were detected were called heterozygous. Five MICA STR polymorphisms were identified in this study: A4, A5, A6, A9 and A5.1. All analyses of the MICA gene including the design of primers, amplification, and next generation sequencing were conducted in the University of Minnesota Genomic Center (UMGC).

### Laboratory measurements of s-MICA levels

s-MICA plasma levels were assessed using the Luminex Bead-based assay in the Cytokine Reference Laboratory (University of Minnesota) following the manufacturer’s instructions (R&D Systems, Minneapolis, MN), as discussed in detail in our previous study[31]. Only samples with soluble MICA levels greater than 2.0 pg/mL were considered positive and included in the study, based on the detection limit of the ELISA assay. The protocols for the laboratory measurements and genetic analyses are available at dx.doi.org/10.17504/protocols.io.2cngave.

### Statistical analysis

The demographic, lifestyle and other characteristics of pancreatic cases and controls were compared using a t-test for continuous variables, and a chi-square test for categorical variables. The main focus of our analysis was the A5.1 polymorphism, because it is a functional variant that encodes a truncated MICA protein. The A5.1 polymorphism was modeled in two ways: as a dominant model (i.e. categorized as having no A5.1 allele or having at least one copy of the A5.1 allele) and as an additive model, i.e. presented as a three-level variable: no A5.1(X/X), heterozygous A5.1 (X/A5.1), or homozygous A5.1 (A5.1/A5.1).

To address the non-normal distribution of s-MICA levels, geometric means of s-MICA were used to compare s-MICA levels across A5.1 genotypes for pancreatic cancer cases and controls. To conduct a multivariate analysis, s-MICA values were log transformed and general linear regression was used to estimate the relative risk (RR) and 95% confidence intervals (CI) for mean s-MICA levels across MICA alleles. The multivariate models were adjusted for pancreatic cancer risk factors including age, sex, education, smoking status, alcohol consumption, and diabetes status.

Unconditional logistic regression was used to calculate the odds ratio (OR) and 95% CI for pancreatic cancer associated with the MICA A5.1 polymorphism using the dominant and additive genetic models. In similar fashion to our general linear regression models described above, all logistic regression models were adjusted for age, sex, education, smoking status, alcohol consumption, and diabetes status. In an additional analysis, we adjusted for the s-MICA levels to test whether the MICA A5.1 is associated with pancreatic cancer via the s-MICA.

Finally, we conducted three exploratory analyses. First, we examined whether the association between the MICA A5.1 genotype and pancreatic cancer risk differed by age category (stratified at the median age), sex, education, diabetes history, smoking history and alcohol consumption. Interaction was examined on a multiplicative scale by including the product of the A5.1 variant and the variable of interest. Our second exploratory analysis examined the association between four other MICA STR polymorphisms (A4, A5, A6, and A9) and pancreatic cancer risk using unconditional logistic regression. Similar to A5.1, the MICA STR polymorphisms were categorized using an additive genetic model (i.e. being homozygous, heterozygous or having no copy of a particular allele) and a dominant genetic model, i.e. categorized as having no allele or at least one copy of the specified allele (A4, A5, A6, or A9). Further, we evaluated the association of eight cancer-associated MICA-SNPs (rs1051792, rs1051794, rs1051798, rs1051799, rs1063635, rs1131896, rs1131898, rs1140700) with pancreatic cancer risk in our study[24,27–30] as well as the association between a functional SNP MICA-129 (rs1051792) and s-MICA levels, as it has been previously reported to modulate s-MICA shedding [24,27].

The *p-*value for statistical significance was determined *a priori* at p<0.05, and at p<0.10 for interaction on the multiplicative scale. All statistical analyses were conducted using SAS software (version 9.4; SAS Institute, Cary, NC).

## Results

**Fig 1:**
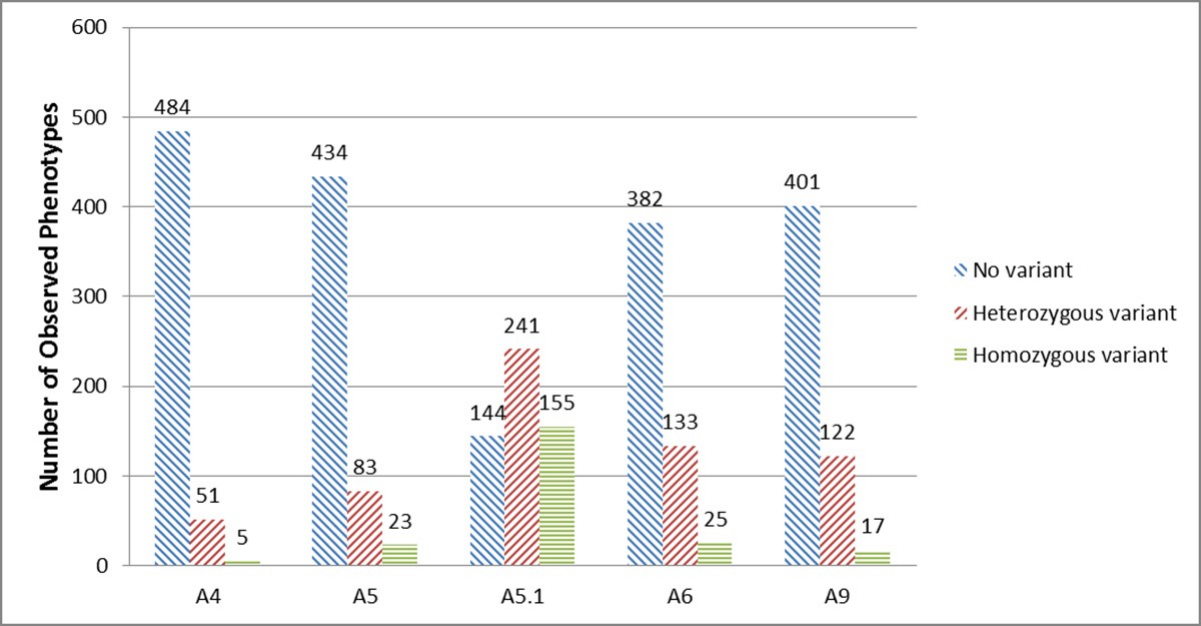
Distribution of MICA STR polymorphisms (A4, A5, A5.1, A6, A9). MICA genotypes were coded as not having the allele, being heterozygous or being homozygous for the allele if the participant possessed 0, 1 or 2 allele copies for the polymorphism, respectively (additive model).

The median age of the 540 participants in this study was 68 years, and 44% were female (**Table 1)**. Participants with pancreatic cancer were more likely to smoke (18% vs. 12%; p = 0.02) and report a history of diabetes (21% vs 8%, p = <0.01), but they were less likely to drink alcohol (11% vs. 18%; p = <0.01) or be college educated (47% vs 60%, p = <0.01) compared to controls **(Table 1)**. The observed frequency of the MICA A5.1 allele was 73% (n = 396), with 155 participants being homozygous and 241 participants being heterozygous for MICA A5.1 (**Fig 1**).

**Table 1:** Distribution of study participant characteristics among pancreatic cancer cases and controls.

Participant’s characteristics	Categories	Number of participants	Cases N (%)	Controls N (%)	P-value
Sex	Female	239	46 (38.02)	193 (46.06)	
	Male	301	75 (61.98)	226 (53.94)	0.11
Age*	<68y	284	68 (56.20)	216 (51.55)	
	>68y	256	53 (43.80)	203 (48.45)	0.41
Smoking status	Never	237	42 (34.71)	195 (46.54)	
	Former	231	57 (47.11)	174 (41.53)	
	Current	72	22 (18.18)	50 (11.93)	0.01
Alcohol consumption (Servings/week)	0	243	67 (62.62)	176 (42.62)	
	1–6	189	28 (26.17)	161 (38.98)	
	≥7	88	12 (11.21)	76 (18.40)	0.01
Diabetic status	No	483	96 (79.34)	387 (92.36)	
	Yes	57	25 (20.66)	32 (7.64)	<0.01
Education	Less than College	229	63 (52.07)	166 (39.62)	
	College Educated	311	58 (47.93)	253 (60.38)	<0.01

* Age was stratified at median

### Association between s-MICA levels and the MICA A5.1 polymorphism

**Fig 2:**
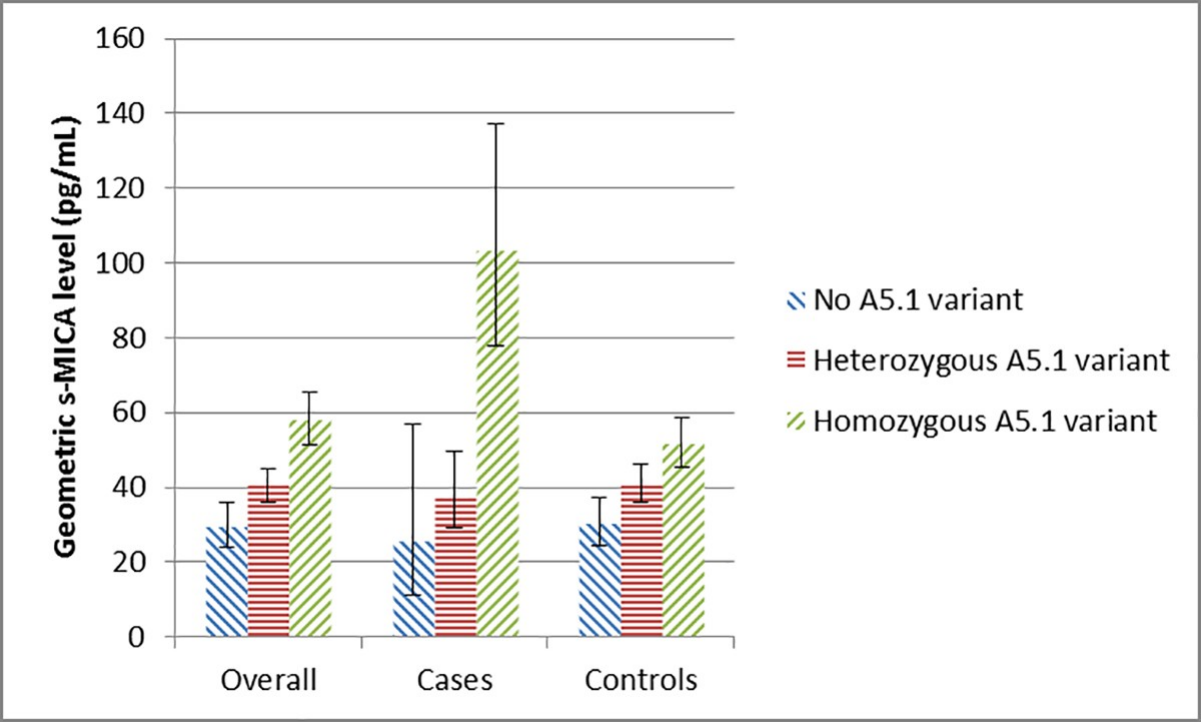
Distribution of s-MICA levels by MICA A5.1 polymorphism genotype. Unadjusted geometric means for s-MICA levels are presented for each group of participants having 0, 1 or 2 A5.1 allele copies for the polymorphism, respectively (additive model). The error bars represent the lower and upper 95% CI for the unadjusted s-MICA geometric mean values.

To evaluate the association between the A5.1 genotype and s-MICA levels in the underlying population, we first examined unadjusted s-MICA levels among controls. Compared to those who did not have a copy of the A5.1 allele, controls with one and two copies of the A5.1 allele had consistently higher unadjusted s-MICA levels (pg/mL), and the highest levels were observed among those with two A5.1 alleles (mean (95% CI) = 30.2 (24.5–37.1), 40.9 (36.1–46.2) and 51.5 (45.4–58.5), respectively). The patterns were similar among all study participants and among pancreatic cancer cases, with higher values being observed for pancreatic cases (**Fig 2**). Similar trends remained in all three groups (controls, cases and total study sample) after adjustment for confounders in both dominant and additive models (**Table 2**). In the additive models with MICA A5.1 as a three-level variable, there was a dose-response relationship between s-MICA levels and the number of copies of the A5.1 allele. Compared to controls without a copy of the A5.1 allele, controls with one copy had 1.24 (95%CI: 0.97–1.59) times greater mean s-MICA levels, and controls with two copies had 1.38 (95%CI: 1.06–1.80) times greater mean s-MICA levels (**Table 2**).Similar dose response relationships were also observed among cases and the total study sample, and the strongest association between s-MICA levels and the number of copies of the A5.1 allele was observed in pancreatic cancer cases.

**Table 2:** Association between circulating MICA levels (s-MICA) and the genotype distribution of the MICA A5.1 polymorphism (dominant and additive models).

MICA Genotype	Number of Participants	s-MICA Levels	Relative ratio of adjusted	p-value
		(Geometric Mean)	geometric means (95% CI)^a^	
MICA A5.1 Polymorphism (Dominant Model)				
Total cohort				
X/X	144	50.10 (38.91–64.50)	Reference	
X/A5.1 or A5.1/A5.1	396	67.66 (60.46–75.72)	1.35 (1.05–1.74)	0.02
Pancreatic cancer cases				
X/X	25	39.41 (14.89–104.27)	Reference	
X/A5.1 or A5.1/A5.1	96	66.77 (48.42–92.06)	1.69 (0.62–4.62)	0.30
Controls				
X/X	119	52.66 (41.34–67.08)	Reference	
X/A5.1 or A5.1/A5.1	300	67.56 (60.08–75.96)	1.28 (1.01–1.63)	0.04
MICA A5.1 Polymorphism (Additive Model)				p-trend
Total cohort				
X/X	144	49.07 (38.20–63.03)	Reference	
X/A5.1	241	60.84 (53.33–69.41)	1.24 (0.96–1.61)	
A5.1/A5.1	155	79.03 (69.05–90.46)	1.61 (1.23–2.11)	<0.01
Pancreatic cancer cases				
X/X	25	38.66 (17.31–86.32)	Reference	
X/A5.1	65	45.18 (30.84–66.21)	1.17 (0.50–2.72)	
A5.1/A5.1	31	98.02 (71.03–135.27)	2.54 (1.07–6.02)	<0.01
Controls				
X/X	119	52.11 (40.88–66.42)	Reference	
X/A5.1	176	64.86 (56.76–74.10)	1.24 (0.97–1.59)	
A5.1/A5.1	124	72.15 (62.22–83.68)	1.38 (1.06–1.80)	0.01

^a^ Adjusted for age (continuous variable), sex (males vs. females), education (no college vs. some college), smoking status (never, former or current), alcohol consumption (no consumption, 1–6 servings per week or 7+servings per week), diabetes status (yes vs. no)

### Association between the MICA A5.1 polymorphism and pancreatic cancer risk

In a multivariate model, having at least one copy of the MICA A5.1 allele was associated with an increased risk of pancreatic cancer (**Table 3**). The multivariate-adjusted OR was 1.91, 95%CI: 1.05–3.48, for cases compared to participants without an A5.1 allele. After additional adjustment for s-MICA level, the A5.1 genotype was no longer associated with pancreatic cancer risk (OR = 1.91, 95%CI: 1.05–3.48). When the A5.1 genotype was modeled as a three-level variable, there was no dose-response relationship between A5.1 genotype and estimated pancreatic cancer risk (**Table 3**).

**Table 3:** Association between the genotype distribution of the MICA A5.1 polymorphism (dominant and additive models) and pancreatic cancer risk.

MICA Genotype	Cases	Controls	OR (95%CI)	p-value
MICA A5.1 Polymorphism (Dominant Model)				
Model 1^a^				
X/X	25	119	Reference	
X/A5.1 or A5.1/A5.1	96	300	1.91 (1.05–3.48)	0.02
Model 2^b^				
X/X	25	119	Reference	
X/A5.1 or A5.1/A5.1	96	300	1.48 (0.77–2.86)	0.24
MICA A5.1 Polymorphism (Additive Model)				p-trend
Model 3^a^				
X/X	25	119	Reference	
X/A5.1	65	176	2.02 (1.11–3.68)	
A5.1/A5.1	31	124	1.51 (0.71–3.22)	0.06
Model 4^b^				
X/X	25	119	Reference	
X/A5.1	65	176	1.57 (0.82–3.02)	
A5.1/A5.1	31	124	1.05 (0.45–2.46)	0.94

^a^ Adjusted for age (continuous variable), sex (males vs. females), education (no college vs. some college), smoking status (never, former or current), alcohol consumption (no consumption, 1–6 servings per week or 7+servings per week), and diabetes status (yes vs. no)

^b^ Multivariate model ^a^ additionally adjusted for s-MICA levels

### Exploratory analyses

We examined whether the association between the MICA A5.1 genotype and pancreatic cancer risk differed by age (below and above 70 years), sex, education (no college vs some college), diabetes history, smoking history (never, former and current) and alcohol consumption (no alcohol consumption, 0–6 servings per week and 7 or more servings per week). Although we did not find statistically significant interactions (all p-values were >0.10), having at least one copy of the A5.1 allele was associated, significantly, with pancreatic cancer among women: OR = 2.58 (95% CI: 1.11–5.96), but not men: OR = 1.24 (95%CI: 0.51–3.02) (p-for interaction = 0.43). Of note, in the subset of participants without diabetes, those with at least one copy of the MICA A5.1 allele remained at an apparent increased risk of pancreatic cancer, OR = 2.01 (, 95% CI: 1.04–3.89) implying that the observed association between A5.1 allele and pancreatic cancer was not driven by diabetes (**S1 Table**). In our analysis of additional MICA STR polymorphisms, we did not observe any significant association between the A4, A5, A6, or A9 MICA STR polymorphisms and pancreatic cancer risk (**S2 Table**). Finally, in the analyses of cancer-associated MICA SNPs, s-MICA levels were greater among participants with the MICA-129 Val / Val genotype (**S3 Table**), but this association was only statistically significant for pancreatic cancer cases with the MICA-129 Val / Val genotype who had 3.06 times great mean s-MICA levels [95% CI: 1.62–5.77)] compared to cases with MICA-129 Met/Val genotype. No pancreatic cancer cases with detectable s-MICA level had the MICA-129 Met / Met genotype (**S3 Table**). No SNPs were statistically significantly associated with pancreatic cancer risk, most likely to limited sample size but there were indications of an association for several SNPs **(S4 Table)**.

## Discussion

In this population-based case-control study, we found that having at least one copy of the MICA A5.1 allele was associated with an increased risk of pancreatic cancer. We also showed that participants with the MICA A5.1 allele had elevated circulating levels of s-MICA, in both controls and pancreatic cancer cases, with higher levels in the cases. This finding is in line with our previous findings of an association between elevated s-MICA levels and increased pancreatic cancer risk[31]. In addition, we reported that the association between the MICA A5.1 allele and pancreatic cancer disappeared after adjusting for s-MICA levels implying that the s-MICA was on causal pathway between MICA A5.1 allele and pancreatic cancer.

Consistent with our reports, several investigators have demonstrated that the MICA A5.1 allele appears to modulate cancer susceptibility in both candidate gene and genome-wide association studies (GWAS). A recent Swedish GWAS found that the MICA A5.1 allele was associated with a 42% increase in cervical carcinoma risk [20]. Similarly, a case-control study of a Han Chinese reported that MICA A5.1 was associated with a 47% increase in hepatocellular carcinoma [26], while in a Japanese case-control study, having MICA A5.1 allele was associated with a 37% increase in oral squamous cell carcinoma risk, and significantly higher s-MICA levels in cases than in healthy controls[22]. In contrast, null associations were reported in studies that examined associations between the MICA A5.1 polymorphism and colorectal cancer[40], gastric cancer[41], and melanoma[42].

Our findings of higher s-MICA levels and increased pancreatic cancer risk in participants with the MICA A5.1 polymorphism may be explained by changes in the s-MICA A5.1 protein, as a result of the polymorphism. The A5.1 polymorphism causes a premature stop codon in the transmembrane region of the MICA gene sequence, which results in a truncated MICA protein around its cytoplasmic tail[17,18]. Given that the location of the A5.1 polymorphism is in close proximity to the ADAM 17 cleaving site, the MICA A5.1 protein may be more easily released into the serum compared to the other functional variants of the MICA protein, resulting in higher circulating concentrations of s-MICA [12,16,17]. In line with this mechanism, our study showed that participants who were either homozygous or heterozygous for the A5.1 allele had significantly greater levels of s-MICA, compared to those who lacked the A5.1 allele[17,22]. Our exploratory findings of higher s-MICA levels in participants with the MICA-129 Val / Val polymorphism, another MICA SNPs which modulates s-MICA shedding[24,27], also highlights the importance of MICA polymorphisms on s-MICA shedding.

Further, our findings of a positive association between the A5.1 polymorphism and pancreatic cancer risk are concordant with biological mechanisms explaining MICA shedding into circulation and its interaction with immune cells. In cancer, the interaction between membrane-bound MICA and NKG2D activates anti-tumor NK and T cell responses[6,8,12–16,43]. However, when human tumor cells release s-MICA into circulation, this not only hinders the recognition of MICA expressing tumors by the immune system, but also leads to a systemic downregulation of NKG2D expression on the surface of γδ T cells and αβ CD8+ T cells, thereby further limiting the anti-tumor activity of these immune cells[12,15,44–46]. Participants with at least one copy of the A5.1 allele would express low levels of membrane-bound MICA and higher levels of s-MICA, which may compromise the ability to alert the immune system of neoplastic change and lead to poor or no activation of immune cell response (by NK and CD8+ T cells) against tumor cells[17].

To the best of our knowledge, our study is the first study that documented an association between A5.1 polymorphism and pancreatic cancer. Other strengths of this population-based study include the simultaneous measurement of MICA polymorphisms and s-MICA levels in the same study, a large number of pancreatic cancer cases and controls, and the ability to adjust for potential confounders. However, there are some limitations in the present study. First, the response rate for both cases and controls was slightly less than 60%.This response rate is typical for controls in population-based studies and higher than response rates for cases in many other studies of pancreatic cancer since it is difficult to enroll pancreatic cancer cases due to the very short average survival of patients[33,35,36]. With response rates at this level, selection bias must always be considered; however, we cannot suggest a biologically plausible reason why respondents would differ in a systematic way from non-respondents with regards to MICA genotype and MICA levels either among cases or controls. Lastly, as in any case-control study, recall bias could arise because diabetes and other covariates were self-reported[33,35,36]. However, recall bias would most likely not influence the findings of our study since the MICA genotype and MICA levels were objectively measured and the associations were minimally affected by confounders (as shown in **Fig 2**).

In summary, our results are in line with our hypothesis that participants with the A5.1 MICA polymorphism have higher s-MICA levels and are predisposed to pancreatic cancer development. Although MICA molecules are not specifically tumor associated antigens, they appear to play a functional role in pancreatic cancer. Further studies are warranted to validate our finding and examine this association in multi-ethnic population settings to determine if the mechanisms of action of functional MICA variants are shared among different populations[17]. These findings are important to elucidate the role of immune surveillance in pancreatic cancer, and potentially could lead to devising novel screening strategies for high-risk groups and new treatment for pancreatic cancer patients.

## Supporting information

S1 Table Association between the genotype distribution of the MICA A5.1 polymorphism (dominant and additive models) and pancreatic cancer risk stratified by potential effect modifiers. S1 Table presents the association between the MICA A5.1 genotype and pancreatic cancer risk by strata of age category, sex, education, diabetes history, smoking history and alcohol consumption.
^a^ Adjusted for sex (males vs. females), education (no college vs. some college), smoking status (never, former or current), alcohol consumption (no consumption, 1–6 servings per week or 7+servings per week), diabetes status (yes vs. no).
^b^ Adjusted for age (continuous variable), education (no college vs some college), smoking status (never, former or current) and alcohol consumption (no consumption, 0–6 servings per week or 7+ per week) and diabetes status (yes vs. no).
^c^ Adjusted for age (continuous variable), sex (males vs. females), smoking status (never, former and current) and alcohol consumption (no consumption, 1–6 servings per week or 7+servings per week) and diabetes status (yes vs. no).
^d^ Adjusted for age (continuous variable), sex (males vs. females), education (no college vs some college), smoking status (never, former and current) and alcohol consumption (no consumption, 1–6 servings per week or 7+servings per week).
^e^ Adjusted for age (continuous variable), sex (males vs. females), education (no college vs some college), alcohol consumption (no consumption, 1–6 servings per week or 7+servings per week) and diabetes status (yes vs. no).
^f^ Adjusted for age (continuous variable), sex (males vs. females), education (no college vs some college), smoking status (never, former and current) and diabetes status (yes vs. no).
(DOCX)

S2 Table Association between the genotype distribution of MICA STR polymorphisms (dominant model) and pancreatic cancer risk. S2 Table presents the association between the distribution of other short tandem repeat MICA genotypes (A4, A5, A6 and A9) and pancreatic cancer risk.
^a^ Adjusted for age (continuous variable), sex (males vs. females), education (no college vs. some college), smoking status (never, former or current), alcohol consumption (no consumption, 1–6 servings per week or 7+servings per week), diabetes status (yes vs. no).
(DOCX)

S3 Table Association between circulating MICA levels (s-MICA) and the MICA-129 genotype distribution (additive models). S3 Table presents the association between the distribution of mica129 SNP (rs1051792) genotypes and soluble MICA levels among pancreatic cancer cases and controls.
^a^ MICA-129 polymorphism (rs1051792) was investigated as the change from the Adenosine nucleotide to the Guanine, resulting in a change from the Methionine codon to a Valine codon at codon 129 in exon 3 of the α 2‐heavy chain domain in the MICA gene.
^b^ Adjusted for age (continuous variable), sex (males vs. females), education (no college vs. some college), smoking status (never, former or current), alcohol consumption (no consumption, 1–6 servings per week or 7+servings per week), diabetes status (yes vs. no).
^c^ There were no pancreatic cancer cases with detectable s-MICA and Met-Met genotype.
(DOCX)

S4 Table Association between the genotype distribution of MICA SNPs (additive model) and pancreatic cancer risk. S4 Table presents the association between the distribution of other MICA SNP genotypes (rs1051792, rs1051794, rs1051798, rs1051799, rs1063635, rs1131896, rs1131898, rs1140700) and pancreatic cancer risk.
^a^ The genotypes have been converted to "0" for reference, "1" for heterozygous, "2" for homozygous alternate, and " " for missing. All genotypes with a quality score less than 20 have been set to missing.
^b^ Adjusted for age (continuous variable), sex (males vs. females), education (no college vs. some college), smoking status (never, former or current), alcohol consumption (no consumption, 1–6 servings per week or 7+servings per week), diabetes status (yes vs. no).
(DOCX)
