# Supplementary material for: Association between MICA polymorphisms, s-MICA levels, and pancreatic cancer risk in a population-based case-control study
Source: PLoS One. 2019 Jun 5;14(6):e0217868. doi: 10.1371/journal.pone.0217868 (PMC6550421; doi:10.1371/journal.pone.0217868)
Supplement: S1 Table — presents the association between the MICA A5.1 genotype and pancreatic cancer risk by strata of age category, sex, education, diabetes history, smoking history and alcohol consumption. a Adjusted for sex (males vs. females), education (no college vs. some college), smoking status (never, former or current), alcohol consumption (no consumption, 1–6 servings per week or 7+servings per week), diabetes status (yes vs. no). b Adjusted for age (continuous variable), education (no college vs some college), smoking status (never, former or current) and alcohol consumption (no consumption, 0–6 servings per week or 7+ per week) and diabetes status (yes vs. no). c Adjusted for age (continuous variable), sex (males vs. females), smoking status (never, former and current) and alcohol consumption (no consumption, 1–6 servings per week or 7+servings per week) and diabetes status (yes vs. no). d Adjusted for age (continuous variable), sex (males vs. females), education (no college vs some college), smoking status (never, former and current) and alcohol consumption (no consumption, 1–6 servings per week or 7+servings per week). e Adjusted for age (continuous variable), sex (males vs. females), education (no college vs some college), alcohol consumption (no consumption, 1–6 servings per week or 7+servings per week) and diabetes status (yes vs. no). f Adjusted for age (continuous variable), sex (males vs. females), education (no college vs some college), smoking status (never, former and current) and diabetes status (yes vs. no). (DOCX) [file pone.0217868.s001.docx]

**S1 Table.** **Association between the genotype distribution of the MICA A5.1 polymorphism (dominant and additive models) and pancreatic cancer risk stratified by potential effect modifiers.**

| Potential Modifier | Group 1 | Group 2 | Group 3 | p-interaction |
| --- | --- | --- | --- | --- |
|  | OR (95%CI) | OR (95%CI) | OR (95%CI) |  |
| Age Category^a^ | Under Median Age  (68 years) | Above Median Age  (68 years) |  |  |
| Genotype |  |  |  |  |
| X/X | 1 (Reference) | 1 (Reference) |  |  |
| X/A5.1 or A5.1/A5.1 | 1.51 (0.67 – 3.54) | 2.66 (1.08 – 6.54) |  | 0.19 |
| Gender^b^ | Males | Females |  |  |
| Genotype |  |  |  |  |
| X/X | 1 (Reference) | 1 (Reference) |  |  |
| X/A5.1 or A5.1/A5.1 | 1.24 (0.51 - 3.02) | 2.58 (1.11 - 5.96) |  | 0.43 |
| Education^c^ | No College Education | Some College Education |  |  |
| Genotype | OR (95%CI) |  |  |  |
| X/X | 1 (Reference) | 1 (Reference) |  |  |
| X/A5.1 or A5.1/A5.1 | 3.00 (1.19 - 7.57) | 1.50 (0.64 - 3.51) |  | 0.19 |
| Diabetes Status^d^ | Yes | No |  |  |
| Genotype |  |  |  |  |
| X/X | 1 (Reference) | 1 (Reference) |  |  |
| X/A5.1 or A5.1/A5.1 | 3.16 (0.61 - 16.29) | 2.31 (1.24 - 4.32) |  | 0.44 |
| Smoking Status^e^ | Never | Former | Current |  |
| Genotype |  |  |  |  |
| X/X | 1 (Reference) | 1 (Reference) | 1 (Reference) |  |
| X/A5.1 or A5.1/A5.1 | 1.90 (0.71 - 5.17) | 1.97 (0.77 - 5.02) | 1.68 (0.35 - 8.17) | 0.77 |
| Alcohol Consumption^f^ | 0 servings / week | 1-6 servings / week | ≥ 7 servings / week |  |
| Genotype |  |  |  |  |
| X/X | 1 (Reference) | 1 (Reference) | 1 (Reference) |  |
| X/A5.1 or A5.1/A5.1 | 1.61 (0.74 - 3.52) | 3.46 (1.02 - 11.69) | 0.81 (0.17 - 3.88) | 0.60 |

S1 Table presents the association between the MICA A5.1 genotype and pancreatic cancer risk by strata of age category, sex, education, diabetes history, smoking history and alcohol consumption

^a^ Adjusted for sex (males vs. females), education (no college vs. some college), smoking status (never, former or current), alcohol consumption (no consumption, 1-6 servings per week or 7+servings per week), diabetes status (yes vs. no)

^b^ Adjusted for age (continuous variable), education (no college vs some college), smoking status (never, former or current) and alcohol consumption (no consumption, 0-6 servings per week or 7+ per week) and diabetes status (yes vs. no).

^c^ Adjusted for age (continuous variable), sex (males vs. females), smoking status (never, former and current) and alcohol consumption (no consumption, 1-6 servings per week or 7+servings per week) and diabetes status (yes vs. no).

^d^ Adjusted for age (continuous variable), sex (males vs. females), education (no college vs some college), smoking status (never, former and current) and alcohol consumption (no consumption, 1-6 servings per week or 7+servings per week).

^e^ Adjusted for age (continuous variable), sex (males vs. females), education (no college vs some college), alcohol consumption (no consumption, 1-6 servings per week or 7+servings per week) and diabetes status (yes vs. no).

^f^ Adjusted for age (continuous variable), sex (males vs. females), education (no college vs some college), smoking status (never, former and current) and diabetes status (yes vs. no).
