# Supplementary material for: Association between MICA polymorphisms, s-MICA levels, and pancreatic cancer risk in a population-based case-control study
Source: PLoS One. 2019 Jun 5;14(6):e0217868. doi: 10.1371/journal.pone.0217868 (PMC6550421; doi:10.1371/journal.pone.0217868)
Supplement: S2 Table — presents the association between the distribution of other short tandem repeat MICA genotypes (A4, A5, A6 and A9) and pancreatic cancer risk. a Adjusted for age (continuous variable), sex (males vs. females), education (no college vs. some college), smoking status (never, former or current), alcohol consumption (no consumption, 1–6 servings per week or 7+servings per week), diabetes status (yes vs. no). (DOCX) [file pone.0217868.s002.docx]

**S2 Table. Association between the genotype distribution of MICA STR polymorphisms (dominant model) and pancreatic cancer risk.**

| MICA Genotype | Cases | Controls | OR (95%CI)^a^ |
| --- | --- | --- | --- |
| MICA A4 |  |  |  |
| X/X | 108 | 376 | 1 (Reference) |
| X/A4 or A4/A4 | 13 | 43 | 1.25 (0.57 - 2.73) |
| MICA A5 |  |  |  |
| X/X | 101 | 333 | 1 (Reference) |
| X/A5 or A5/A5 | 20 | 86 | 0.97 (0.53 - 1.76) |
| MICA A6 |  |  |  |
| X/X | 90 | 292 | 1 (Reference) |
| X/A6 or A6/A6 | 31 | 127 | 0.74 (0.44 - 1.24) |
| MICA A9 |  |  |  |
| X/X | 83 | 318 | 1 (Reference) |
| X/A9 or A9/A9 | 38 | 101 | 1.18 (0.40 - 3.51) |

S2 Table presents the association between the distribution of other short tandem repeat MICA genotypes (A4, A5, A6 and A9) and pancreatic cancer risk.

^a^ Adjusted for age (continuous variable), sex (males vs. females), education (no college vs. some college), smoking status (never, former or current), alcohol consumption (no consumption, 1-6 servings per week or 7+servings per week), diabetes status (yes vs. no).
