# Supplementary material for: Association between MICA polymorphisms, s-MICA levels, and pancreatic cancer risk in a population-based case-control study
Source: PLoS One. 2019 Jun 5;14(6):e0217868. doi: 10.1371/journal.pone.0217868 (PMC6550421; doi:10.1371/journal.pone.0217868)
Supplement: S3 Table — presents the association between the distribution of mica129 SNP (rs1051792) genotypes and soluble MICA levels among pancreatic cancer cases and controls. a MICA-129 polymorphism (rs1051792) was investigated as the change from the Adenosine nucleotide to the Guanine, resulting in a change from the Methionine codon to a Valine codon at codon 129 in exon 3 of the α 2‐heavy chain domain in the MICA gene. b Adjusted for age (continuous variable), sex (males vs. females), education (no college vs. some college), smoking status (never, former or current), alcohol consumption (no consumption, 1–6 servings per week or 7+servings per week), diabetes status (yes vs. no). c There were no pancreatic cancer cases with detectable s-MICA and Met-Met genotype. (DOCX) [file pone.0217868.s003.docx]

**S3 Table. Association between circulating MICA levels (s-MICA) and the MICA-129 genotype distribution (additive models)**

| MICA 129 Genotype (rs1051792)^a^ | Geometric Mean s-MICA Levels | RR on the Additive Scale (95%CI) ^b^ | RR on the multiplicative scale (95% CI)^b^ | P-value for trend |
| --- | --- | --- | --- | --- |
|  |  |  |  |  |
| Total cohort |  |  |  |  |
| Met / Met | 41.35 (9.18 - 186.34) | Reference | Reference |  |
| Met / Val | 39.01 (32.04 - 47.49) | 0.06 ((-1.58) - 1.46) | 0.94 (0.21 - 4.31) | 0.94 |
| Val / Val | 78.86 (71.39 - 87.12) | 0.65 (-0.86 - 2.15) | 1.91 (0.42 - 8.62) | 0.41 |
| Pancreatic cancer cases | _ | _ | _ |  |
| Met / Met^c^  Met / Val | 23.38 (10.68 - 51.22) | Reference | Reference |  |
| Val / Val | 71.58 (50.56 - 101.33) | 1.12 (0.49 - 1.75) | 3.06 (1.62 - 5.77) | <0.01 |
| Pancreatic cancer controls |  |  |  |  |
| Met / Met | 38.04 (10.21 - 141.76) | Reference | Reference |  |
| Met / Val | 41.73 (34.43 - 50.51) | 0.09 ((-1.23) - 1.42) | 1.10 (0.29 - 4.13) | 0.89 |
| Val / Val | 76.22 (68.67 - 84.60) | 0.69 ((-0.62) - 2.01) | 2.00 (0.54 - 7.48) | 0.30 |

S3 Table presents the association between the distribution of mica129 SNP (rs1051792) genotypes and soluble MICA levels among pancreatic cancer cases and controls.

^a^ MICA-129 polymorphism (rs1051792) was investigated as the change from the Adenosine nucleotide to the Guanine, resulting in a change from the Methionine codon to a Valine codon at codon 129 in exon 3 of the α 2‐heavy chain domain in the MICA gene.

^b^ Adjusted for age (continuous variable), sex (males vs. females) , education (no college vs. some college), smoking status (never, former or current), alcohol consumption (no consumption, 1-6 servings per week or 7+servings per week), diabetes status (yes vs. no).

^c^ There were no pancreatic cancer cases with detectable s-MICA and Met-Met genotype
