# Supplementary material for: Association between MICA polymorphisms, s-MICA levels, and pancreatic cancer risk in a population-based case-control study
Source: PLoS One. 2019 Jun 5;14(6):e0217868. doi: 10.1371/journal.pone.0217868 (PMC6550421; doi:10.1371/journal.pone.0217868)
Supplement: S4 Table — presents the association between the distribution of other MICA SNP genotypes (rs1051792, rs1051794, rs1051798, rs1051799, rs1063635, rs1131896, rs1131898, rs1140700) and pancreatic cancer risk. a The genotypes have been converted to "0" for reference, "1" for heterozygous, "2" for homozygous alternate, and " " for missing. All genotypes with a quality score less than 20 have been set to missing. b Adjusted for age (continuous variable), sex (males vs. females), education (no college vs. some college), smoking status (never, former or current), alcohol consumption (no consumption, 1–6 servings per week or 7+servings per week), diabetes status (yes vs. no). (DOCX) [file pone.0217868.s004.docx]

**S4 Table. Association between the genotype distribution of MICA SNPs (additive model) and pancreatic cancer risk.**

| Genotype^a^ | Cases | Controls | OR (95%CI) ^b^ | P-value for trend |
| --- | --- | --- | --- | --- |
| rs1051792 A>G |  |  |  |  |
| A/A | 74 | 237 | Reference |  |
| A/G | 39 | 155 | 0.80 (0.29 - 2.20) |  |
| G/G | 8 | 27 | 1.52 (0.54 - 4.24) | 0.06 |
| rs1051794 A>G |  |  |  |  |
| A/A | 73 | 238 | Reference |  |
| A/G | 40 | 155 | 0.78 (0.29 - 2.12) |  |
| G/G | 8 | 26 | 1.38 (0.50 - 3.83) | 0.09 |
| rs1131896 A>G |  |  |  |  |
| A/A | 76 | 244 | Reference |  |
| A/G | 37 | 149 | 0.80 (0.29 - 2.20) |  |
| G/G | 8 | 26 | 1.27 (0.46 - 3.51) | 0.33 |
| rs1131898 G>A |  |  |  |  |
| G/G | 76 | 243 | Reference |  |
| A/G | 37 | 149 | 0.81 (0.30 - 2.22) |  |
| A/A | 8 | 27 | 1.28 (0.47 - 3.53) | 0.38 |
| rs1051798 T>C |  |  |  |  |
| T/T | 51 | 161 | Reference |  |
| T/C | 52 | 191 | 1.28 (0.62 - 2.65) |  |
| C/C | 18 | 67 | 1.74 (0.79 - 3.81) | 0.31 |
| rs1140700 C>T |  |  |  |  |
| C/C | 76 | 239 | Reference |  |
| C/T | 36 | 145 | 0.93 (0.39 - 2.23) |  |
| T/T | 9 | 35 | 1.03 (0.45 - 2.33) | 0.93 |
| rs1051799 G>C |  |  |  |  |
| G/G | 76 | 244 | Reference |  |
| G/C | 37 | 149 | 0.80 (0.29 - 2.20) |  |
| C/C | 8 | 26 | 1.27 (0.46 - 3.51) | 0.33 |
| rs1063635 A>G |  |  |  |  |
| A/A | 53 | 161 | Reference |  |
| A/G | 50 | 190 | 1.23 (0.59 - 2.55) |  |
| G/G | 18 | 68 | 1.79 (0.82 - 3.93) | 0.37 |

S4 Table presents the association between the distribution of other MICA SNP genotypes (rs1051792, rs1051794, rs1051798, rs1051799, rs1063635, rs1131896, rs1131898, rs1140700) and pancreatic cancer risk.

^a^ The genotypes have been converted to "0" for reference, "1" for heterozygous, "2" for homozygous alternate, and " " for missing. All genotypes with a quality score less than 20 have been set to missing.

^b^ Adjusted for age (continuous variable), sex (males vs. females), education (no college vs. some college), smoking status (never, former or current), alcohol consumption (no consumption, 1-6 servings per week or 7+servings per week), diabetes status (yes vs. no).
